# The Outcomes of Primary Scleral Buckling during Repair of Posterior Segment Open-Globe Injuries

**DOI:** 10.1155/2014/613434

**Published:** 2014-06-22

**Authors:** Dan Cohen, Jaime Levy, Tova Lifshitz, Nadav Belfair, Itamar Klemperer, Noam Yanculovich, Boris Knyazer

**Affiliations:** ^1^Joyce and Irving Goldman Medical School, Faculty of Health Sciences, Ben-Gurion University of the Negev, 84101 Be'er-Sheva, Israel; ^2^Department of Ophthalmology, Soroka University Medical Center, Faculty of Health Sciences, Ben-Gurion University of the Negev, P.O. Box 151, 84101 Be'er-Sheva, Israel

## Abstract

*Objective*. To compare visual outcomes of eyes which underwent primary scleral buckling (PSB) treatment during posterior segment open-globe injury (OGI) repair with eyes not treated with PSB. *Methods*. We retrospectively reviewed 38 eyes which underwent a posterior segment OGI repair with no preoperative evidence of retinal detachment (RD) at Soroka University Medical Center (1995–2010). 19 (50%) underwent scleral repair alone (control group) and the other 19 eyes were treated with PSB also (PSB group). We compared visual outcomes in these two groups and rates of subsequent postoperative complications. *Results*. Baseline characteristics of the groups were similar. Compared with the control group, the PSB group had statistically significant lower rates of proliferative vitreoretinopathy (PVR) (5.3% versus 38.4%, *P* < 0.05) and a trend towards lower rates of RD (15.8% versus 41.1%, *P* = 0.1). PSB group eyes had a statistically significant improvement of their best distance visual acuity (BDVA) with lower means of final BDVA-grade (*P* < 0.05) and logMAR vision (*P* < 0.05). Eyes in the control group had no improvement in these parameters. *Conclusion*. PSB procedure during posterior segment OGI repair may decrease the risk of subsequent retinal complications and improve final visual outcome.

## 1. Introduction

Ocular trauma is a common cause of visual impairment and loss in working age patients [1]. In industrialized nations, eye injury has become the most frequent reason for hospitalization of ophthalmologic patients [2]. In the USA alone, there are approximately 2.4 million eye injuries each year, and more than 40,000 result in permanent visual impairment [3].* Open-globe injuries *(OGIs), defined as injuries that include a full-thickness defect in the cornea and or sclera [4], have a reported incidence of between 2 and 6 per 100,000 persons per year [2, 5, 6]. The estimated global incidence rate of OGI is 3.5 cases per 100,000 persons per year, leading to approximately 203,000 OGIs per year worldwide [3].

The OGI classification system [4] has identified important prognostic factors for OGIs and hence classifies them based on the zone of injury, mechanism of injury, the presenting visual acuity, and the presence of a relative afferent pupillary defect (RAPD). Another useful system, the ocular trauma scoring (OTS) system [7], is used to classify ocular trauma and to predict the visual outcome of the injured eye, using parameters of presenting visual acuity, mechanism of injury, presence of endophthalmitis, retinal detachment (RD), and RAPD [7].

While* visual outcomes* of eyes with all-types OGIs may vary from full recovery to complete blindness, it is well recognized that posterior segment (zone II and III injuries) OGIs harbor a worse prognosis [8]. Moreover, these injuries, and particularly zone III injuries (which sometimes involve the macula and optic nerve), frequently correlate with a poor* anatomical outcome* [8–12] despite extensive advances in imaging, instrumentation, materials, and surgical procedures over the decades [8].

The growing understanding that the development of RD in posterior segment OGI might initiate an anatomical destructive series [10, 11, 13–18] has raised the interest in methods that may prevent the development of RD in these injured eyes [19].* Primary scleral buckling* (PSB) of any posterior segment OGI, that is, supporting the retina and vitreous base with an encircling scleral buckle at the time of the primary repair, is one suggested method. Besides the fact that PSB at the time of initial surgery is technically easier than scleral buckling done afterwards, this procedure has the potential to reduce the rates of vitreoretinal traction and of subsequent development of retinal tears and detachment.

Several studies examined the role of scleral buckling in the management of ocular trauma. Some showed that prophylactic scleral buckling at the time of vitrectomy for posterior segment trauma was associated with better outcomes [8–11]. In two retrospective studies [15, 16], PSB at the time of posterior segment open-globe repair was associated with an improved visual and anatomical outcome. However, this surgical procedure is still controversial [19]; therefore we have designed this study to clarify its possible benefits.

## 2. Methods

In this retrospective study we identified and analyzed eyes suffering OGI and underwent primary scleral repair alone or with PSB between 1995 and 2010 at Soroka University Medical Center. The local Ethics Committee at our institution approved this study.

Eyes which had an injury limited to zone I only, less than 3 months of follow-up, evidence of RD (per fundus examination or ultrasonography or at the time of the primary surgery), evidence of endophthalmitis, and eyes whose best distance visual acuity (BDVA) data was missing at the presentation or at the end of the follow-up period were excluded from the study. All included eyes were categorized into two groups according to their PSB status (PSB group and control group).

The chart of each patient was reviewed and evaluated to determine demographic features (age and sex), interval time between trauma and surgery, mechanism of injury, zone of injury, initial BDVA-grade, initial logarithm of the minimal angle of resolution (logMAR) vision score, and a calculated OTS score. Mechanism of injury was classified according to the Birmingham Eye Trauma Terminology [20] as rupture or laceration, and lacerations were classified as penetration, perforation, or IOFB. Zone of injury was defined as zone II or zone III according to the Ocular Trauma Classification Group [4]. The initial Snellen-BDVA was derived from visual tests performed at the time of the patient's admission to our institution. Visual acuity was assessed using a Snellen acuity chart for distance or Jaeger card for near vision. When possible, testing was performed with a pinhole. For eyes without formed vision, the acuity was determined as count fingers (CF), hand motion (HM), light perception (LP), or no light perception (NLP) if the patient was unable to see a bright light source such as the light from an indirect ophthalmoscope. The best assessed visual acuity in these tests was documented as the BDVA. BDVA-grade was divided into five categories according to the Snellen-BDVA, grade 1: 6/6–6/12; grade 2: 6/15–6/36; grade 3: 6/60–CF; grade 4: HM and LP; and grade 5: NLP. The logMAR vision score was calculated by obtaining the logarithm of the reciprocal of the Snellen-BDVA for vision better than or equal to 6/60. Otherwise, the following conversion was used: FC = 1.6, HM = 2.0, LP = 2.5, and NLP = 3.0 logMAR units. OTS score was calculated according to the calculation system that was developed by Kuhn et al. [7].

The outcomes evaluated in this study were final BDVA-grade, final logMAR vision score, rates of RD, rates of proliferative vitreoretinopathy (PVR), rates of ocular hypertension, and number of subsequent surgeries. The data regarding the outcomes was obtained from the outpatient record of each patient at his latest follow-up visit in our institution, with all included patients having minimum of 3 months of follow-up. BDVA-grade was determined and logMAR vision score was calculated the way it was described above for the initial data. Data regarding number of subsequent surgeries was collected as an ordinal parameter divided into one surgery, two surgeries, or multiple (three or more) subsequent surgeries during follow-up period. The development of a postoperative RD and PVR was noted for each patient.

All operations in our institute were performed by two well experienced retinal surgeons (Itamar Klemperer and Nadav Belfair) with similar surgical approaches. After detailed examination and exploration of the eyes, primary scleral wound repair was performed in the operating room with nonabsorbable suture such as 8-0 nylon. Cases of vitreous incarceration were treated by scissors or with vitrectomy till complete release of vitreal remnants. Afterwards, in PSB group eyes, an encircling 3.5 to 4.0 mm solid silicone exoplant was placed with four scleral fixation sutures in the middle of each oblique quadrant. Finally, patients received broad spectrum intravenous antibiotics for at least 72 hours after surgery.

### 2.1. Statistical Analysis

Statistical analysis was carried out using SPSS for Windows (version 19.0.0, SPSS Inc., Chicago, IL, USA). When comparing the baseline characteristics and the outcomes between the two groups, continuous and ordinal variables were analyzed using Mann-Whitney test, and binary variables were analyzed using *χ* ^2^ trend test or Fisher's exact test. When comparing between the final and the initial BDVA within each group, analysis was done using Wilcoxon signed-rank test. A *P* value of 0.05 or less was accepted as statistical significance.

## 3. Results

Basic data was collected for 85 patients with OGI, of whom 47 were excluded, with the main reasons for exclusion being injuries limited to zone I and lack of recorded data. Full data was obtained for 38 patients with posterior segment OGI who underwent primary scleral repair at Soroka University Medical Center and had no evidence of RD at their presentation on fundus examination, ultrasonography, nor on the surgery itself. Of the 38 patients' eyes, 19 eyes (50%) underwent PSB during the primary scleral repair (PSB group), and 19 eyes (50%) underwent the repair alone (control group).

Baseline demographic and clinical characteristics of the patients were compared and are presented in Table 1. No statistically significant differences were found. All the eyes underwent the repair less than 24 hours from the injury, with a similar interval time period between the trauma and the surgery (*P* = 0.65, Table 1). All patients were treated with systemic and topical antibiotics, and they all underwent brain and orbits computed tomography scans to exclude intracranial and intraorbital pathology.

Visual and anatomic outcomes in the two groups are presented in Table 2. The mean follow-up for the PSB group was 20.5 months and for the control group it was 16 months, so that time between initial surgery and most recent BDVA assessment was equivalent in the two groups (*P* = 0.33). Compared with the control group, eyes with PSB had statistically significant lower rates of PVR (5.3% versus 38.4%, *P* = 0.03). The PSB group had also lower rates of RD (15.8% versus 41.1%), but this difference did not reach statistical significance (*P* = 0.1). Although not statistically significant, the analysis also suggested a trend toward improved visual outcome in the PSB group. Eyes in the PSB group had a lower mean final BDVA-grade (2.37 versus 2.89, *P* = 0.2) and a lower mean logMAR score vision (1.10 versus 1.38, *P* = 0.3). The number of the subsequent surgeries was similar between the groups (*P* = 0.6). At the end of the follow-up period, no patient suffered from phthysis of the injured eye, nor sympathetic ophthalmia. No cases presented with posttraumatic endophthalmitis.

**Figure 1:**
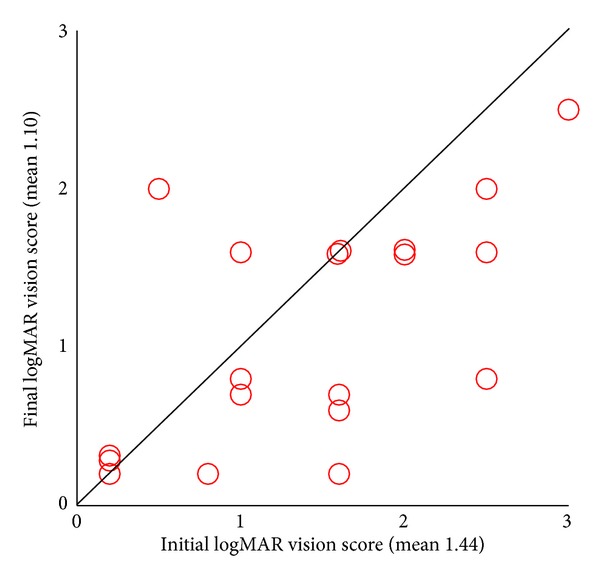
Distribution of final logMAR vision score in correspondence with the initial one in the PSB group (*n* = 19). *P* value = 0.04. PSB: primary scleral buckling; logMAR: logarithm of the minimal angle of resolution. Analysis was done using Wilcoxon signed-rank test.

**Figure 2:**
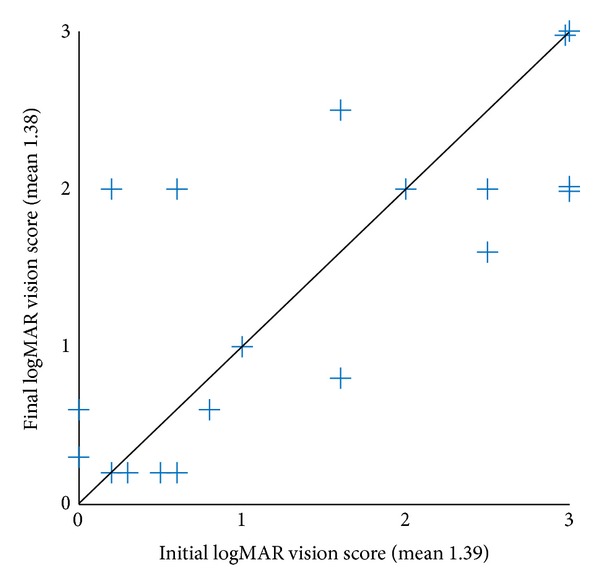
Distribution of final logMAR vision score in correspondence with the initial one in the control group (*n* = 19). *P* value = 0.73. LogMAR: logarithm of the minimal angle of resolution. Analysis was done using Wilcoxon signed-rank test.

Another analysis we made was comparison between the final BDVA and the initial BDVA within each group. This analysis revealed that eyes in the PSB group had a statistically significant improvement in their BDVA while the eyes in the control group had no change in their BDVA. Comparing the final and the initial BDVA-grade in the PSB group (Table 3) showed statistically significant improvement of 0.58 steps in the mean BDVA-grade of these eyes, from mean initial BDVA-grade of 2.95 to final BDVA-grade of 2.37 (*P* = 0.02), with only 1 of the 19 eyes having deteriorated final BDVA-grade. LogMAR vision score analysis of the PSB group eyes (Figure 1) showed again a statistically significant improvement, from initial mean score of 1.44 to a final mean score of 1.10 (*P* = 0.04). The same statistical analysis for the control group eyes found no improvement of their BDVA. Comparison between the final and the initial visual parameters showed statistical similarity for both BDVA-grade (*P* = 0.96, Table 4) and logMAR vision score (*P* = 0.73, Figure 2), as is also indicated with a fairly even distribution above and beneath the line in Table 4 and in Figure 2.

## 4. Discussion

Posterior segment OGIs are associated with poor visual and anatomical outcomes [8–12]. A series of anatomical events following an OGI has been described in animal models [13] and appears to follow an analogous series in humans. This series begins with the development of retinal breaks or rhegmatogenous detachment which tend to occur weeks to months following the injury. A subsequent PVR might evolve with the formation of a fibrocellular membrane and a possible further traction [14]. Theoretically, prevention of RD formation in an open-globe injured eye may halt that anatomical course and improve its prognosis. PSB procedure may offer such prevention but still is not considered a consensus [19] and lacks an evidence-based benefit.

In this study, we compared two groups of eyes with posterior segment OGIs which had no evidence of RD at their presentation and underwent their initial surgery at Soroka University Medical Center, Be'er-Sheva, Israel, between 1995 and 2010. The PSB group underwent PSB in addition to their primary scleral repair, and the control group underwent posterior segment open-globe repair alone.

In our study, 41.1% of the eyes in the control group developed RD after the initial open-globe repair, whereas only 15.8% of the eyes in the PSB group developed a RD. The rate of RD in our control group eyes is comparable to the rate reported in the ophthalmologic literature for all-OGI eyes (40–57%) [10, 11, 15–17], and the rate of RD in our PSB group is lower than this reported rate. RD rate in our PSB group is comparable to that reported in the PSB group of the matched study published by Arroyo et al. (26%) [16]. Such trend is also seen in the analysis of PVR development in our study, which occurs in 10–45% of injured eyes according to previous literature [17, 18]. In our study, the control group had a PVR rate of 38.4% (within the reported range), whereas the PSB group had PVR rate of 5.3% only.

Comparing the outcomes between the PSB and the control groups after average follow-up greater than 18 months, our findings suggest that the addition of PSB at the time of the initial surgery decreases the incidence of subsequent PVR (*P* < 0.05) and may decrease the rates of subsequent RD (*P* = 0.14). Moreover, it may improve the final BDVA of these injured eyes (*P* = 0.22 for final BDVA-grade, and *P* = 0.36 for final logMAR vision). These findings suit the assumption that PSB procedure may improve anatomical and visual outcomes.

Another finding of our study can be derived from comparison of initial to final BDVA within each group. It can be concluded that the addition of PSB improves BDVA while having the repair alone does not change BDVA. This finding can be also derived from the results reported by Arroyo et al. [16] although it was not analyzed statistically there.

Comparison of baseline characteristics between both groups in the study found no statistically significant differences (Table 1). However, one nonsignificant difference strengthens our conclusions regarding the advantages of PSB procedure. The PSB group had worse initial BDVA. Given that poor initial BDVA predicts poor prognosis in OGI cases, one could predict that the PSB group would have worse visual outcome. However, our results showed that PSB group eyes had actually better visual outcome.

Our study has still some limitations. First, this study is a retrospective study which is weaker than a prospective one. Furthermore, the study did not include a case-case matching since an attempt we made to match the eyes according to their baseline injury characters ended with a very low number of couples and a very low potential statistical power. Secondly, owing to limited patient documentation, the two groups could not be compared at few baseline parameters (such as presence of RAPD, choroidal detachment, vitreous haemorrhage, cataract, length of laceration, etc.). Thirdly, this study has finally included relatively few eyes, hence low statistical power. Only one difference between the groups (in PVR) reached statistical significance.

## 5. Conclusion

Our study suggests that adding PSB procedure during posterior segment OGI repair in eyes without evidence of RD at the presentation decreases the risk of subsequent PVR, may decrease subsequent RD, and may improve BDVA when compared to repair alone. In addition, final BDVA is improved compared to the initial BDVA when PSB is added, while it may not change when having the repair alone. These results suggest that PSB in these eyes may have the potential to alter the expected anatomic consequences of their eye injury and hence may improve their overall outcome. Given the high incidence of ocular trauma in the working age population and its poor prognosis, a larger retrospective study, a meta-analysis, or a randomized clinical trial is warranted to more definitively examine the role of PSB.

## Figures and Tables

**Table 1:** Baseline demographic and clinical characteristics of PSB and control patients.

	PSB group *n* = 19	Control group *n* = 19	*P* values
Male	16 (84.2%)	18 (94.7%)	0.60
Mean age (yr)	34.53 ± 15.45	36.84 ± 15.75	0.58
Mean lag time between trauma and surgery (hr)	12.53 ± 8.48	12.80 ± 7.21	0.65
Rupture	7 (36.8%)	7 (36.8%)	1.00
Laceration	12 (63.2%)	12 (63.2%)	1.00
Penetration	2 (10.5%)	6 (31.6%)	0.23
Perforation	0 (0%)	2 (10.5%)	0.49
IOFB	10 (52.6%)	6 (31.6%)	0.19
Zone II	5 (26.3%)	6 (31.6%)	0.72
Zone III	14 (73.7%)	13 (68.4%)	0.72
Mean initial BDVA-grade	2.95 ± 1.13	2.84 ± 1.54	0.78
Mean initial logMAR vision score	1.44 ± 0.85	1.39 ± 1.15	0.77
Mean OTS score	2.68 ± 0.82	2.68 ± 1.37	0.90

PSB: primary scleral buckling; IOFB: intraocular foreign body; BDVA: best distance visual acuity; OTS: ocular trauma score; logMAR: logarithm of the minimal angle of resolution.

Continuous and ordinal variables were analyzed using Mann-Whitney test. Binary variables were analyzed using *χ* ^2^ trend test or Fisher's exact test.

**Table 2:** Outcomes for PSB and control patients.

	PSB group *n* = 19	Control group *n* = 19	*P* values
Mean follow-up (mo)	20.47 ± 14.93	16.00 ± 13.01	0.33
Mean final BDVA-grade	2.37 ± 1.07	2.89 ± 1.45	0.22
Mean final logMAR vision score	1.10 ± 0.73	1.38 ± 0.98	0.36
RD	3 (15.8%)	7 (41.1%)	0.14
PVR	1 (5.3%)	5 (38.4%)	0.03
Ocular hypertension	0 (0%)	1 (5.8%)	0.47
Subsequent surgeries	2.11 ± 0.88	1.95 ± 0.97	0.60

PSB: primary scleral buckling; BDVA: best distance visual acuity; logMAR: logarithm of the minimal angle of resolution; RD: retinal detachment; PVR: proliferative vitreoretinopathy.

Continuous and ordinal variables were analyzed using Mann-Whitney test. Binary variables were analyzed using *χ* ^2^ trend test or Fisher's exact test.

**Table 3:** Comparison between the final and the initial BDVA-grade in the PSB group (*n* = 19). *P* value = 0.02.

Final BDVA-grade (mean: 2.37)	**5**					
	**4**		1		1∗	1
	**3**			3∗	3	
	**2**			4	1	
	**1**	3∗	1	1		
		**1**	**2**	**3**	**4**	**5**

	Initial BDVA-grade (mean: 2.95)					

PSB: primary scleral buckling; BDVA: best distance visual acuity.

Analysis was done using Wilcoxon signed-rank test.

**Table 4:** Comparison between the final and the initial BDVA-grade in the control group (*n* = 19). *P* value = 0.96.

Final BDVA-grade (mean: 2.89)	**5**					2∗
	**4**	1	1	1	2∗	2
	**3**			1∗	1	
	**2**	1	1∗	1		
	**1**	3∗	2			
		**1**	**2**	**3**	**4**	**5**

	Initial BDVA-grade (mean: 2.84)					

PSB: primary scleral buckling; BDVA: best distance visual acuity.

Analysis was done using Wilcoxon signed-rank test.
